# Medicinal Cannabis and Consumer Vulnerability in Australia: A Nexus of Policy and Market Factors

**DOI:** 10.1111/hex.70176

**Published:** 2025-02-10

**Authors:** Katrina Gething, Daniel Erku, Paul Scuffham

**Affiliations:** ^1^ Centre for Applied Health Economics, School of Medicine and Dentistry Griffith University Brisbane Queensland Australia; ^2^ Menzies Health Institute Queensland Griffith University Gold Coast Queensland Australia

**Keywords:** consumer vulnerability, medicinal cannabis, medicinal cannabis policy

## Abstract

**Introduction:**

Following the 2016 legalization of medicinal cannabis (MC) in Australia, significant barriers have led patients to seek unregulated cannabis for therapeutic use. This study examines consumer (patient, carer and family) submissions to a senate inquiry on these barriers to understand how future policy might better reflect patient needs and facilitate access to regulated MC.

**Methods:**

Sixty submissions from patients (*n* = 44), their caregivers or family members (*n* = 16) were coded using NVivo 12 software and thematically analysed. The findings were presented narratively using a consumer vulnerability framework.

**Results:**

The analysis identified three primary barriers to accessing regulated MC: (1) Health practitioners' reluctance to prescribe MC, hindering prescription access, (2) High costs associated with MC and its access process, disproportionately affecting low‐income consumers and (3) Dependence on imported MC products, leading to shortages and necessitating product substitutions that incur additional costs and bureaucratic hurdles. Despite these barriers, consumers demonstrated resilience by educating themselves about MC, planning for prescription needs and forming support networks. Patients also turned to illicit MC markets.

**Conclusion:**

The study reveals significant barriers to regulated MC access in Australia, highlighting the complex challenges consumers face. The reliance on unregulated sources of MC not only poses legal and health risks but also underscores the urgent need for policy reforms. By addressing the identified barriers, such as alleviating the costs associated with MC and improving approval processes and ensuring product availability, policymakers can better meet consumer needs and facilitate safer access to regulated MC.

**Patient or Public Contribution:**

The materials of this document analysis were patient and public submissions to a government enquiry into barriers to a health technology. By providing submissions, both patients and the public were actively engaging in the development of health policy.

## 1 Introduction

The provision and use of medicinal cannabis (MC) is a complex policy issue [1]. In most countries, the regulation of MC is linked with the prohibition of cannabis. Cannabis control occurred in the early 19th and 20th centuries when governments around the world ratified several international drug control initiatives that had declared cannabis a drug with a high risk of abuse [2]. Current attitudes toward cannabis have ushered a shift in the cultural zeitgeist, particularly with regard to the largely unsubstantiated medical benefits of cannabis [3, 4]. In response, many governments are having to reconcile the legal provision of MC with embedded drug policy and national medicine frameworks. The Australian experience of MC regulation is a case in point.

Australia's *Narcotic Drugs Act 1967* (The Act) provides a framework for the control of narcotics and the availability of these drugs for medical and research purposes in accordance with the Single Convention on Narcotic Drugs [5]. In 2016, Australia's federal parliament passed amendments to *The Act* to allow controlled cultivation of and access to cannabis for medicinal or scientific purposes through a single national licensing scheme [5]. Although legal, MC is largely an unapproved therapeutic good as most MC products do not meet the evaluation criteria for approval. Special access pathways for unapproved therapies in response to the needs of particular people or circumstances have been established since enactment of The Therapeutic Goods Act 1989 [6, 7]. As such, an established mechanism for accessing unapproved MC in Australia existed and was utilised upon MC legalisation.

The ready availability of illicit cannabis and backyard formulations of MC may have scuppered the success of Australia's MC policy. A survey conducted 2 years after an Australian MC policy was introduced found that providing legal access to MC had little impact and patients were opting to access illicit cannabis to treat a range of complaints [8]. Evidence of Australian patients' struggle to access legal forms of MC led to a 2019 Senate inquiry on ‘Current barriers to patient access to medicinal cannabis’ [9] hereafter referred to as ‘The Inquiry’.

The Inquiry aimed to review several key aspects of Australia's MC framework, including its regulatory appropriateness, jurisdictional access, regulatory and financial barriers and the effects of these on patients' mental and physical health. Leveraging submissions to The Inquiry, this study aims to: (1) uncover the perspectives of patients, families, and carers on MC use and access policy and (2) examine how future policies can more accurately reflect the needs of consumers with the goal of moving patients from illicit markets to prescribed MC.

## 2 Methods

We follow the READ approach to document analysis [10]. Steps include (i) readying the materials, (ii) extracting data, (iii) analysing the data and (iv) distilling the findings.

### 2.1 Readying the Materials

The first step of the READ approach to document analysis requires the researcher to establish the source, nature and number of the documents to be analysed.

On 14 November 2019, the Australian Senate referred an inquiry into MC access to the Senate Community Affairs References Committee, with a report expected by the 26 March 2020. A media release was issued on the 15 November 2019 publicizing the call for submissions stipulating these were to be received by the 17 January 2020 via an online submission site. An online guide suggested structuring submissions around the Terms of Reference for The Inquiry (available as Supporting Information). Authors could ask for their submissions to be confidential. No demographic details other than jurisdiction was recorded.

The Inquiry attracted 146 submissions from a diverse group of stakeholders. This study specifically analysed submissions from patients, highlighting the narratives of individuals who have accessed or have attempted to access legal or illicit MC (*n* = 45). Submissions from caregivers and family members were included (*n* = 17), providing a comprehensive view of the consumer experience at that time. For this analysis, submissions that did not address the Terms of Reference of The Inquiry and did not discuss barriers to access and usage were excluded (*n* = 2), resulting in a total of 60 submissions being considered for in‐depth analysis.

### 2.2 Extracting Data

The second step of the READ process requires the researcher to determine what and how data are extracted from the documents and includes adopting a theoretical or conceptual framework as well as practical decisions on the coding software or processes. In this study, the analytical lens of consumer vulnerability (CV) was applied. CV is a concept that encapsulates the experiences of powerlessness and dependence consumers may face in a particular market due to various conditions or circumstances [11]. Models of CV typically aim to: (1) identify factors that contribute to or mitigate the experience of vulnerability within a market, (2) describe the experiences of CV and (3) illustrate the actions taken by consumers and other stakeholders to address CV [12, 13, 14, 15]. Contributors (or antecedents) to CV span a broad range of factors including individual characteristics (e.g., age, socioeconomic status, illness); social expectations (e.g., norms, stereotypes); industry practices (e.g., pricing and marketing strategies, discrimination), as well as wider forces such as geographic location, legal and regulatory frameworks [16]. The current study provides a brief outline of individual characteristics to provide context but gives greater weight to those antecedents to which policy can be directed such as industry and policy features.

The critical components of the actual consumer experience of vulnerability are a lack of personal control in the market and the potential for consumer harm from market interaction [11, 13, 14]. Consumer and stakeholder responses to vulnerability in the marketplace are the final component of CV models [14].

Files of consumer submissions were imported into NVivo (version 1.5.2). Categories for coding were predefined according to the common elements of CV models (antecedents of CV, experience of vulnerability, responses to CV).

### 2.3 Analysing the Data

The third phase of the READ process requires authors to question both the data and the source. Methodology is established at this point.

Data were read with reference to a CV framework. The level of analysis within the framework where relevant include micro‐level (i.e., subjective perspective), meso‐level (i.e., transactional perspective) and macro‐level (i.e., regulatory and industry) perspective. Tracts of text were coded and collated according to the predefined categories and level of analysis.

Previous studies have used frequency counts or some other quantitative method to aid analysis [17]. Quantitative cut‐off points were used in the current study to gauge the strength of support amongst the 60 submissions for a theme within a CV component. Themes with recorded comments from at least 20% of viable consumer submissions were read closely and similarities and contradictions recorded. Key themes were those that referenced comments from at least 80% of consumer submissions.

The source of the documents was assessed for credibility. Submissions were highly subjective. Author agenda was apparent and included resolving MC access issues and reducing MC process and product costs among others. Submissions often included emotive accounts of illness, financial and psychological stress and criminal offences. Significant bias was apparent with most authors expressing strong beliefs in MC efficacy as a treatment for a wide range of conditions and symptoms despite a dearth of clinical evidence to support the claims.

### 2.4 Distiling the Findings

The final phase of the READ approach is to refine the findings, preserve particularly illustrative examples within the documents and express the results as a narrative that satisfies the aims of the research. Quotes were included in the narrative when they exemplified the theme being discussed. Submissions tended to be lengthy (between 1 and 26 pages) and quotes used were usually edited down for ease of reading. All attempts were made to capture the essence of the consumer's meaning in these edits and the interested reader is directed to the original, publicly available transcripts for the full context.

## 3 Results

The following analysis is ordered according to the key activities in CV research. Consumer submissions were subjective. Where appropriate, neutral descriptions of applicable MC policy accompany consumer accounts to provide a balanced narrative.

### 3.1 Antecedents to Vulnerability

**Figure 1:**
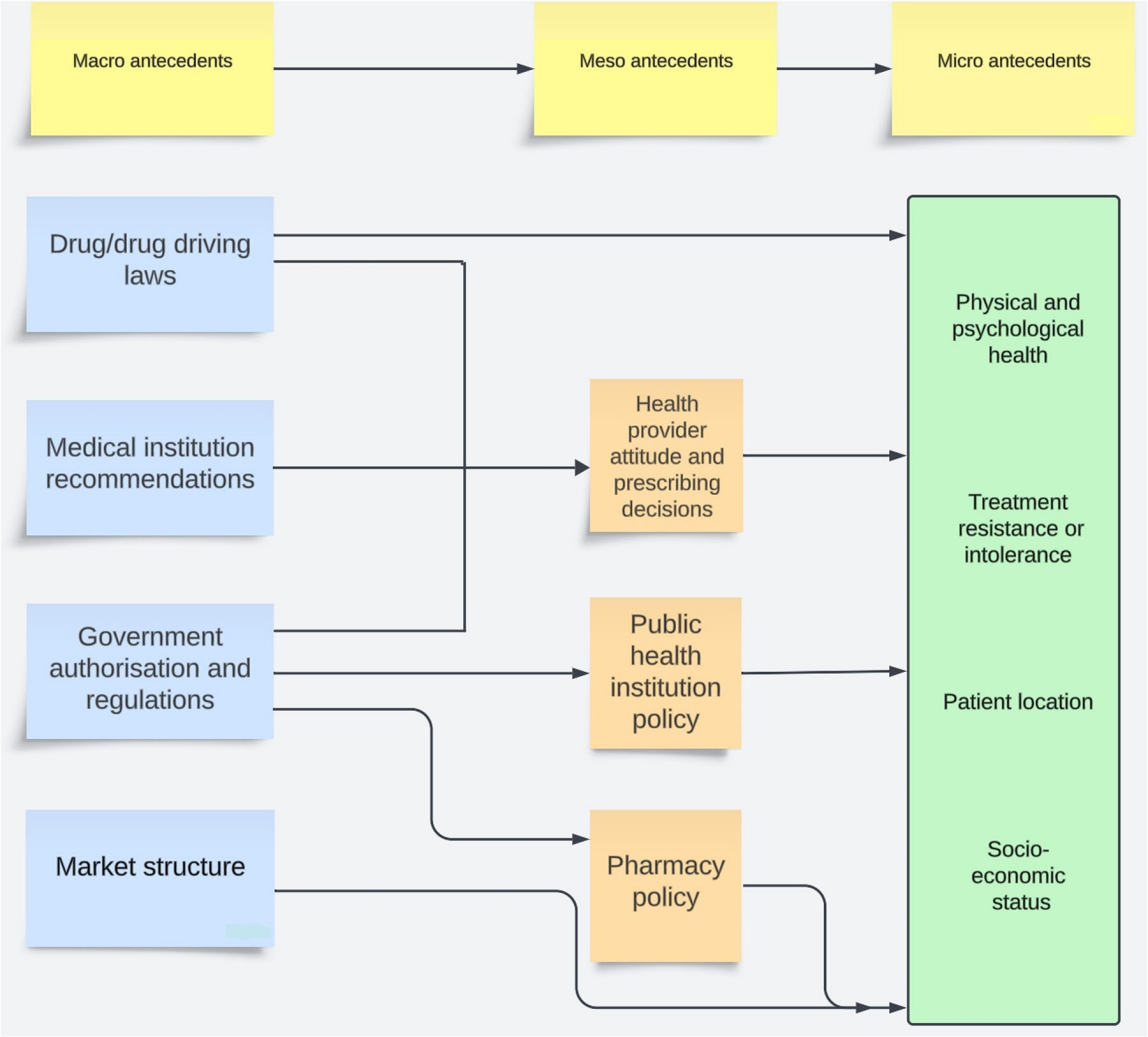
Antecedents to consumer vulnerability in the Australian medicinal cannabis marketplace.

CV in the MC marketplace largely stemmed from a confluence of meso‐ and macro‐level features that created pressure on consumers predisposed to CV. A description of micro‐, meso‐ and macro‐ antecedents follows, and a graphical presentation is provided in Figure 1.

#### 3.1.1 Micro‐Level Antecedents

Early studies of CV focused on how consumer characteristics could predispose a consumer to vulnerability [18, 19, 20]. An obvious consumer characteristic in the current context is physical health. Ill health is the motivation for pursuing MC as a therapy and inspection of submissions revealed patients were experiencing chronic and/or severe to dire illness including intractable and severe forms of epilepsy, symptoms of multiple sclerosis, autoimmune diseases, PTSD and other psychological complaints, cancer, non‐cancer chronic pain and rare life‐threatening diseases. A further defining consumer characteristic was treatment resistance or medication intolerance. Standard approved treatments either did not work or were not able to be endured and MC was perceived by consumers to be the final therapy option for the condition and/or symptoms being experienced.

After almost 18 years on pharmaceutical antiseizure medications T developed such severe toxicity that 2 GP's and a specialist advised the ceasing of all such medications. T spent most days bedridden… We were desperate to try anything that might give him some kind of meaningful life. Cannabis was our only option and hope.

(Carer/family member 12)

Understandably, access to an efficacious and tolerable medicine increases in importance for patients experiencing severe illness, predisposing them to CV when access to that medicine is restricted.

Membership to socioeconomic group influenced CV. Serious illness has a financial impact and many respondents (both patients and carers) relied on government support payments because their illness or full‐time caring duties precluded them from gainful employment. The price and associated costs of MC were therefore felt more keenly by a large subset of consumers.

Geographical location also impacted the likelihood of CV being experienced. Limited numbers of health providers (HPs) work in rural and regional Australian towns and medical centres tend to limit the admission of new patients because of an inability to accommodate them. Subsequently, patients living in these areas experienced difficulty in accessing a HP who would prescribe MC.

#### 3.1.2 Meso‐ and Macro‐Level Impacts

A nexus of institutional (macro‐level) and transactional (meso‐level) forces combined to create barriers at every consumer touchpoint in the journey to MC access and use. Foremost were consumer experiences with HPs. In Australia, MC must be prescribed, affording HPs the role of gatekeepers to legal MC access. In the years immediately after legalization in 2016, consumers had trouble finding a HP to prescribe MC. Consumers linked HP reticence toward MC with their unfamiliarity with it as a therapy and described experiencing prejudice and animosity during medical appointments.

I asked a number of local (rural) GPs about accessing cannabis [for my mother], [they] were not interested in discussing it… So after a further pain‐filled wait until her next 6 monthly appointment, my mother asked the specialist again about trying cannabis. He [said] “If you ask again you may find yourself looking for another doctor!”. He had nothing to offer in its place…everything else had already been tried or ruled out because of allergy.

(Carer/family 14)

Several explanations for HP reticence to consider MC as a therapy have been proposed. Foremost is its status as an unapproved therapy. Australia's leading medical fraternities initially cautioned HPs in prescribing MC citing the dearth of scientific evidence to support its use in most clinical circumstances [21].

Our Neurologist and Paediatrician were willing to script us many meds [that] gave our daughter horrendous side effects, but they didn't have the confidence to script CBD [a form of MC] as they continually advised that there was not enough evidence.

(Carer 4)

HPs amenable to prescribing MC were discouraged by the regulatory body's patient authorisation process. Health specialists may become authorized prescribers of MC for a class of patients though this process requires endorsement from a specialist college. All other authorizations require an HP to apply for approval on behalf of each individual patient. Further complicating the issue for prescribers was the two‐tier application system. Patient approval for MC is required from both the federal and jurisdictional health departments. The special access application processes were described by respondents as challenging for HPs to navigate.

…my long‐time general practitioner and my integrative Dr were supportive of me trying it but we're unaware that they could prescribe medicinal cannabis easily. (They both felt the government hoops were protracted and complicated to attempt personally).

(Consumer 15)

The intersection between application processes and Australia's reliance on imported MC put further pressure on HPs. Supply was inconsistent, necessitating brand and/or product substitutions. Australia's MC policy required HPs to name the product they intended to prescribe to a patient. Product substitutions therefore required new applications, further burdening HPs and adding to patient costs and delaying prescriptions.

L has now used medicines in Australia from 5 licenced producers and 3 licenced bulk importers. To date 7 of the 8 companies have experienced out of stock periods or an inability to maintain supply or consistent supply. One company advised they would not continue making the same formulation and two companies have stopped shipping another product into the country completely.

(Carer/family 15)

Regulations around the storage and provision of MC products exacerbated delays in patient access. Pharmacies must adhere to regulations on MC storage and ordering and may only order MC for a named consumer with an authorized prescription. Subsequently, inventory was not held by pharmacists. The ordering‐MC‐on‐demand practices of pharmacies coupled with the vagaries of a market relying on imported product created frequent delays in filling prescriptions.

Barriers to use at an institutional level impacted consumer ability to medicate with MC. Experiences with hospital stays for patients using MC were variable and consumer descriptions of their care suggest an institutional MC policy in the years immediately after legalization had not been established.

…I attempted to provide the nurse with approvals and notifications however the nurse would not take them and would not permit L to have his prescribed medicines and would not allow me to leave L's cannabis oil or cannabis flower and vaporiser for administration through the night should he require them after any seizure episodes, or for his morning dose.

(Carer/family 15)

There are evident issues with allowing the use of MC during hospital stays. It is probable that the restrictive S8 drug classification of MC at the time impacted MC use in institutional settings. Institutional policy usually specifies that a patient's own supply of S8 medications may not be used in a public health institution, and that an S8 medication be prescribed, supplied and administered to the patient by the hospital during their stay [22]. Further, smoking or vaporizing raw flower in a hospital ward is unlikely to be condoned for health reasons and due to smoking in public laws.

The decision to use MC therapy was influenced by drug driving laws. It is illegal to drive in Australia under the influence of trans‐Δ9‐tetrahydrocannabinol (THC) (an intoxicating component of cannabis). The deleterious effects of THC on driving performance such as increased lane deviation, and cognitive abilities such information processing and divided attention have been established, though dosage effect and impairment duration is yet to be determined [23]. Prescribing HPs are required to inform patients of driving restrictions and consumers who disclose a need to drive are prescribed Cannabidiol (CBD)‐based MC (which has no intoxicating effect). Drug driving laws were a particularly salient issue for rural and regional patients who rely on being able to drive to access basic services and to be a part of the community.

… Living in a regional area with very little public transport I am not in a position to give up my licence and stop driving to access cannabis… you are asked if your licence is important to you and if you answer ‘yes’ your prescription choices are then reduced to the products without THC present. For those living with debilitating Chronic Pain this choice reduces the chance that you will be prescribed a product that is likely to work.

(Consumer 17)

### 3.2 Experiences of Vulnerability

**Figure 2:**
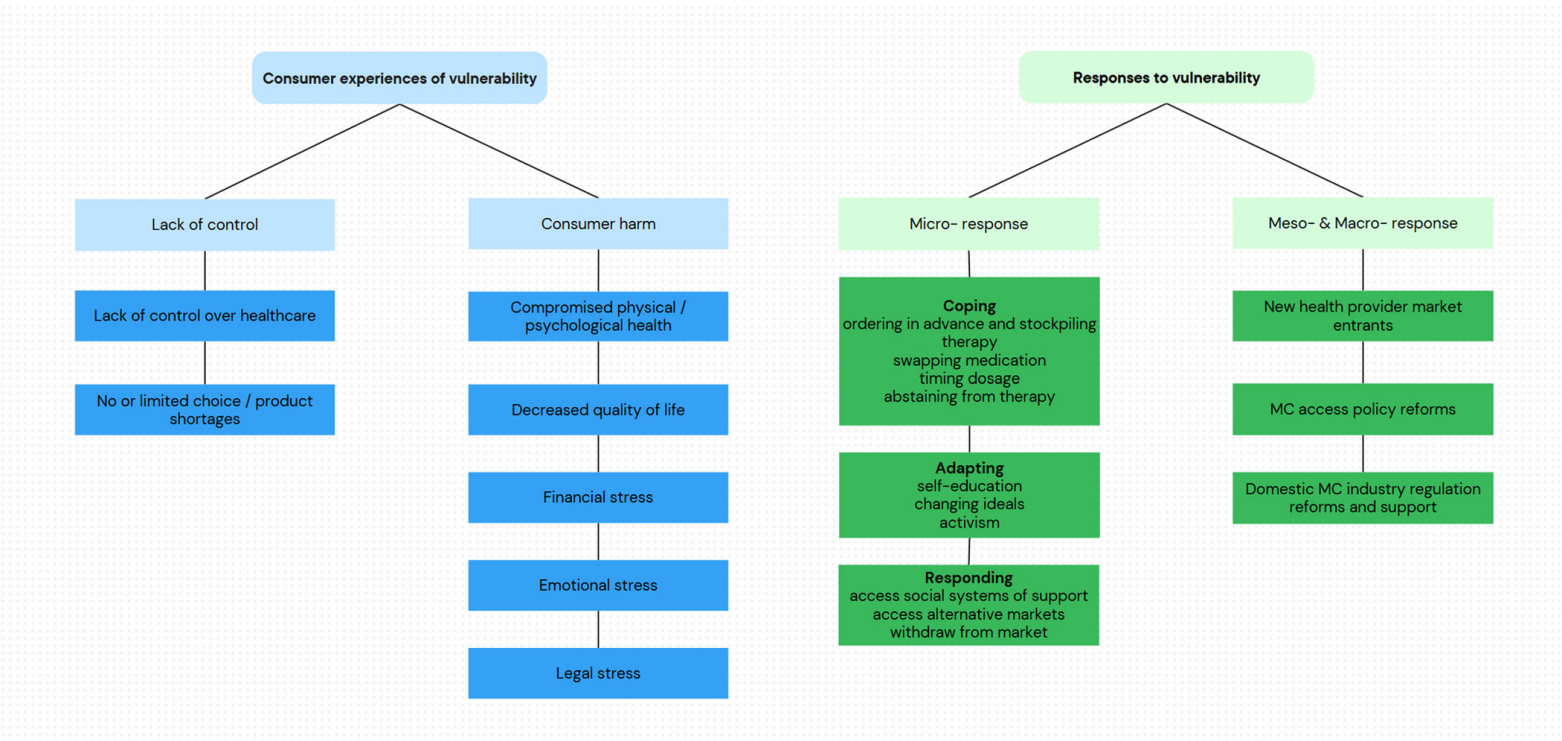
Experiences of consumer vulnerability and responses to that vulnerability in the Australian medicinal cannabis marketplace.

CV is defined in terms of two key factors: a lack of personal control [11], and the potential for consumer harm [11, 13]. Figure 2 summarizes MC consumer experiences of CV.

The intersection of meso‐ and macro‐influences previously discussed restricted consumer control of their healthcare and their ability to interact in the MC service system. This was a phenomenon felt acutely by rural and regional patients.

The only pain specialist I have access to in Darwin refused to look at the [regulatory] approval and the [jurisdictional] process for prescribing medicinal cannabis.

(Consumer 35)

Choice in the market was also limited. Regulatory requirements around specifying a named product in applications and restrictions on advertising prevented consumers from being able to search for lower‐cost MC options.

[The supplier] said their protocol is not to inform potential purchases of the prices… you cannot contact a number of suppliers to compare prices. I told [the supplier] how much I am paying for the medicine that I receive from another company and they said that they can supply that item cheaper but will not mention the price so I can compare them against each other.

(Consumer 23)

Consumers who were unable to access MC therapy experienced harm because their symptoms were not treated or because they needed to revert to approved medicines that were intolerable or ineffective. According to submissions, MC provided relief for a range of symptoms. In some cases of rare, intractable and life‐threatening illness, MC was perceived as the only therapy to provide quality of life.

As a blind pensioner I can not afford this life changing drug. …the CBD and THC oil literally changed my life, for the better…, unfortunately as i can no longer afford the CBD oil, I'm back on opiods and anxiety medication which make me sick.

(Consumer 25)

Consumers who were approved for a MC prescription experienced harm in the form of financial stress. The unapproved and therefore unsubsidised status of MC meant consumers, the majority of which were receiving some form of government benefit, could not afford the therapy or the processes for procuring a prescription. One submission revealed that treatment costs, including initial appointments and associated application fees, MC therapies and the required follow‐up appointments had depleted their retirement account.

A further consequence of not being able to access MC for this cohort is deteriorating psychological health for patients, and stress and grief for their families and carers.

The last weeks of David's life were so very hard for him and for me … There was nothing that I could do to ease his pain. After David died, I called his oncologist to advise him of David's death. He told me he still had not received permission to prescribe Medicinal Cannabis for David.

(Carer/family 16)

### 3.3 Responses to Consumers Experiencing Vulnerability

This section details the micro‐ (consumer), meso‐ (transactional) and macro‐(regulatory/industry) responses to CV. Figure 2 summarizes the responses to CV.

#### 3.3.1 Micro‐Level Responses

Consumer responses to vulnerability are categorized as coping, adapting and responding [24]. We define coping as engaging in behaviours to manage experiences of vulnerability [14]. We use the word adaptation to mean a change by which a person becomes better suited to their circumstances. We define consumer response as the consumer's actions to reduce or eliminate vulnerability in the marketplace [14].

Vulnerable consumers developed coping strategies to minimise their experiences of vulnerability. Patients strategized to overcome shortages in MC supply by ordering in advance of and in excess to their needs. Consumers with budgetary concerns reduced their intake of MC to increase the time between filling prescriptions.

[Our son] was prescribed with a dose of 6.5 mL per day based on his current weight. Due to the high cost of medicinal cannabis, my husband and I can only afford to obtain a regular dose of 0.6 mL per day, and even affording this dose is a financial struggle. For our son to have his recommended dose, it would cost us $52 per day, $1612 per month, $19,344 per year…We cannot provide our son the medication that could at the least, change his life and maybe even save it!

(Carer/family member 1)

Patients who needed to drive a vehicle swapped THC dominant medications for CBD, abstained from medication before driving or timed their THC‐based medications for night only. Some patients withdrew from the MC market.

I'll need to make two visits to my chemist. Firstly, to drop off the script and in a few days’ time another to pick up the medication as it does not get delivered to me at home. This means that I must not take my medication for almost a week so that I can drive.

(Consumer 3)

Conservative consumers adapted by undergoing personal transformations to reduce the cognitive dissonance experienced by considering MC as a therapy. These consumers pursued MC therapies as a last resort. The success they experienced with the therapy led them to reconsider their personal ideals. Conservative respondents became MC proponents and activists rather than detractors, resolving their identity crises by incorporating their changed views about MC into their values, thereby staying true to the core of their beliefs.

We knew that as Christians we needed to be transparent and declare publicly what we were doing. We also wanted to educate people on the benefits derived from using medicinal Cannabis. By providing experienced knowledge we hoped others would not make the stupid, wilful mistakes we had made in condemning medicinal Cannabis.

(Carer/family 12)

Consumers also adapted in response to CV by educating themselves about the cannabinoid system and phytocannabinoids, as well as learning how to cultivate cannabis and manufacture MC. The objective of self‐education was three‐fold: to understand if an alternative to conventional therapies existed, to meet HP gaps in MC knowledge, and finally to self‐supply MC.

Consumer' response to CV was to develop social systems of support. Patient‐specific or MC‐specific communities provided advice and facilitated access to MC, either legally or illicitly. Members were gifted MC to trial or medicine was shared when supply was unstable or unaffordable.

We were given around $1000 of medical cannabis oil (around 6–8 weeks supply)—if it were not for the generosity of friends, there is no way we could afford this (T is on a disability pension; my parents are on carers pensions and have no capacity to work as they both care for T 24 h a day/7 days a week.

(Carer/family 12)

Communities also provided advice on navigating the patient approval system. The following is an extract of an email sent to a member of such a community by a mother whose son has been diagnosed with multiple conditions for which MC was purported to be a treatment.

I just wanted to feed back to you that since your email, I was able to get my son into see Dr xxx and today I collected his CBD oil… Thank you so much for your assistance, if you hadn't of put me in touch with Dr xxxx, I don't know how we would have found him.

(Communication sent to Consumer 27)

Many consumers responded to the barriers to legal MC access by accessing alternative markets, specifically recreational cannabis. Recreational cannabis was simpler and quicker to access and less costly than legal MC. Consumers were not required to attend and pay for multiple medical appointments and regulatory approval, costs were transparent, and supply was constant. Some respondents manufactured forms of MC from recreational cannabis.

I have had no choice but to source illegal Cannabis product to keep my daughter alive. Once I source the product I then convert the dry plant material into a liquid medication; which I produced in the kitchen of our family home.

(Carer/family 6)

Respondents experiences with and views of having to access recreational cannabis varied. Some consumers were comfortable with sourcing unregulated cannabis while others experienced stress. The quote below is one patient's account of the process and is indicative of the risky trade‐offs consumers made for quick, cost‐efficient and regular supply.

…despite [the black market] being quick (30 min quick), there is no quality control… if the intent is a medical one where you require consistent and predictable dosage, the [black market] is completely unreliable. Anyway, I approached one of the underground Uberised drug dealers—who asked if I'd like any LSD tabs or MDMA pills while he was at it.

(Consumer 39)

‘Green market’ sources of unregulated MC were more palatable to a subset of consumers. The Green market refers to illicit MC sourced from MC community members, or self‐proclaimed healers. Healers were self‐taught MC specialists that produced unregulated MC products at a ‘reasonable’ cost or by donation specifically for community members.

These are the ‘quiet Australians’ that have resorted to access via a more compassionate and better educated ‘green market’ which currently provides a level of care and support that makes the medical profession look wretched and the political system look laughable.

(Carer/family 11)

Consumers who could not afford the costs of illicit MC responded by growing their own cannabis. All green market healers had been prosecuted under cannabis drug laws and consumers of illicit MC experienced stress associated with the prohibition of cannabis.

It is alleged Mr [T] had 107 plants which he was juicing for his two daughters—who suffer Crohn's Disease when he was raided by police. He explained in simple terms: “The ideal juicing is 30 mL, 3 times a day: 2 girls, 180mls a day—that's a lot of plant material.”

(Consumer 13)

#### 3.3.2 Meso‐ and Macro‐ Level Response to CV

A demand‐service gap was evident in the provision of MC that was filled by medicinal cannabis clinics (MCC). MCCs responded to consumer needs in four ways. First, they provided access to HPs who would evaluate the patient, determine their eligibility and prescribe MC for qualifying and authorised patients. Secondly, MCC medical staff have knowledge about the endocannabinoid system, cannabinoids and their therapeutic uses. Third, their exclusive focus on the provision of MC helped them navigate the regulatory process and facilitate access for consumers. Finally, rural and regional patients could access MCC services remotely and postal delivery was offered by some clinics.

While MCCs facilitated access to MC to patients who could afford their services, they also contributed to experiences of financial vulnerability. MCC are profit‐based, and marketing practices were evident. Respondents described MCC ‘lock‐in’ practices. Consumers described having to register with a clinic before an appointment was offered, signalling switching costs. Australian regulations stipulate only one HP may prescribe MC for any given patient. Switching to a different clinic or doctor would necessitate additional expenses for appointments and a new application to the regulatory body.

My first Cannabis for medical use application cost $200 for the Cannabis clinic consultation, then $90 for the [regulatory] approval application, then required check‐ups at $75ea. The actual Cannabis cost $168.50 for 5 grams… At this rate, I'd be spending about $25,818 per year just in medication, plus all the required consultations, checkups and further approvals. That's $10,000 more than I earn…

(Consumer 18)

Macro‐level responses to CV in the period after The Inquiry are addressed in the Discussion.

## 4 Discussion

The provision of MC presents a challenge for policy makers. Clinical evidence for most MC therapies is yet to be established. Yet, public consensus is for MC to be legally available to those that need it [25, 26], and patient‐reported outcomes suggest the therapy holds value [27, 28]. However, there are cautions to be made. Cannabis is a drug with the potential for abuse and its use may impair cognitive functions [23]. THC has been linked to certain cardiac conditions [29] and research outcomes point to its role in the development of psychiatric disorders [30], particularly in young people [31]. An effective policy would reflect public preferences and employ safeguards to public health while also considering the welfare of patient populations that may benefit from MC. The current study is concerned with the final point and found that a confluence of MC policy and market characteristics created significant barriers to access and negatively impacted the welfare of patients, their carers and families. Consumers primarily responded by accessing alternative MC markets. The goal of this study was to gain an understanding of how future policy might better reflect consumer needs with the objective of moving patients from accessing illicit to regulated forms of MC.

### 4.1 Primary Barriers to MC Access

As the gatekeepers to health care HPs were the foremost barrier to the provision of MC. Consumer descriptions of the attitudes and behaviours of HPs with regard to MC prescription are in contrast to the findings of two systematic reviews which found general support for the use of cannabinoid‐based therapies amongst medical practitioners [32, 33], though this support was condition‐specific in Australia [34]. Regardless, it was the nexus of HP reluctance, the costs of access to an unapproved therapy and the irregular supply of MC therapies that effectively created the MC service system failure that prompted The Inquiry.

### 4.2 Implications for Policy

Patients' primary need was to access affordable MC in a timely, reliable and uncomplicated manner. Patients also expressed a desire to be able to drive a vehicle while using MC containing THC and to legally cultivate cannabis for their own therapeutic use. We consider the policy implications of each of these needs and wants and discuss solutions already implemented by the Australian government as well as policy alternatives.

It is reasonable to assume that a healthy domestic MC cultivation and manufacturing industry would realize Australian patients' need to access a reliable supply of affordable MC therapies. Regulation changes designed to foster a domestic industry are being made incrementally [35], and other broad government industry initiatives will assist the government's goal of Australia becoming a major manufacturer of MC products [36, 37]. However, the Australian industry is yet to meet its domestic needs. In 2020, imported products supplied 90% of MC consumed in Australia [38]. Furthermore, the total quantity of domestically cultivated stock has been steadily decreasing since 2021 while the export of domestic MC stock has been increasing [39]. Conceivably, increasing government support of the domestic industry's export opportunities may help local companies increase production capacity and hasten economies of scale—ultimately allowing the industry to flourish and then reliably supply the domestic market.

Consumers believe the path to affordable MC lay with government subsidies. This scenario is unlikely. The Australian Government subsidizes the cost of approved medicines under the Pharmaceuticals Benefits Scheme (PBS). Just two MC therapies, Sativex (for symptoms of multiple sclerosis) and Epidyolex (to treat seizures) are approved though neither of these has been recommended for reimbursement under the PBS. Other countries with a prescription model of MC access offer examples of low‐cost compassionate access for specific patients. The Named Patient Pharmaceutical Assessment Policy is the New Zealand government's process for funding unapproved treatments for individuals whose clinical circumstances are life‐threatening or extremely severe [40]. In Italy, MC prescriptions originating from public health systems and for specific pathologies warrant significant government reimbursement [41, 42]. Payment relief for Australian patients, however, is likely to come from the private health insurance sector. Certain Australian private health insurers offer some form of coverage of unapproved therapies accessed via special pathways, and the Australian government subsidizes health insurance for low‐income patients. In Europe, German and Czech patients rely on health insurance rebates to ameliorate the costs of MC.

Australian patients want uncomplicated access to MC. The pathways chosen to allow access to MC were originally established to provide patient access to unapproved therapies in extenuating circumstances. The large demand for MC put considerable pressure on these established but seldom used processes. Policy changes have since occurred, and patient authorisation procedures for MC have been simplified. Arguably, the greatest change has been the recognition of MC as a broad range of therapies. MC therapies are now categorised according to the ratio of the cannabinoids THC and CBD. MC therapies with limited psychotropic effect (i.e., CBD‐dominant) are now included in an ‘established history of use’ pathway for specific conditions and patient approval processes have been simplified for authorised prescribers. Also, applications now refer to a category of MC rather than a named product, negating the requirement for additional applications if a product substitute in the same category is required. HPs must continue to justify the prescription of MC therapies with intoxicating levels of THC when applying for patient approval.

A segment of patients sought the legal ability to grow their own cannabis. Most submission authors clearly preferred to access legal MC but felt the barriers to access gave them no choice but to source unregulated product. A smaller number of respondents articulated a preference for unregulated product. Patient autonomy, ideas of natural versus chemical, and patient experience with recreational cannabis have previously been implicated in this preference [43, 44], and were noted during this analysis but were outside the scope of the study to consider any further. Australian jurisdictional governments have intermittently considered liberal cannabis laws. At the time of writing, households in the Australian Capital Territory were able to cultivate a small number of cannabis plants for their own use. Future research might consider the impacts of decriminalisation on the MC and illicit cannabis markets and the impacts of using home cultivated cannabis on patient health and wellbeing.

Consumers using THC‐based MC need to drive, particularly those living in rural and regional areas of Australia. Restrictions on driving were a significant barrier to access with around a third of consumers voicing concerns. Patient‐led suggestions for alternative drug driving laws include exemptions for patients with a MC card, patient discretion based on packaging warning labels and replacing zero tolerance laws with more lenient actions such as police field sobriety tests. There is a need for more research on dosage effect and impairment duration before alternative policies can be considered.

### 4.3 Contributions to CV Literature

This study's focus on response to CV contributes to CV scholarship. Previous research has likened vulnerability in the marketplace to powerlessness and dependence and a reduced capacity to act [11, 12, 15, 45]. The current study reveals ways in which consumers regain autonomy and power both within the market and outside of the market. The coping strategies used by patients, such as delaying prescription dispensing have previously been noted as a cost‐saving measure [46]. Patient stockpiling behaviours to cope with therapy shortages is a recognised strategy, most recently observed during the COVID‐19 pandemic [47]. Like past CV studies, consumers also coped by creating and accessing community member support [48, 49]. Not only were information and product shared, but the emotional support provided by members increased the psychological capacity to remain in the market. Community support also allowed consumers to exit the market and access new, albeit illicit markets.

Consumers psychologically adapted to their circumstances. The finding that conservative consumers educated themselves about MC to dispel psychological discomfort about cannabis‐based therapies supports previous patient research that found changing ideologies came about by educating oneself about personal healthcare, leading to a feeling of personal responsibility thereby reducing the experience of vulnerability [50].

Consumers responded to their vulnerability by accessing new markets. The MC market is distinct in that an alternative market can be readily accessed by consumers denied access to a legal one [1]. Unregulated forms of MC were considered cheaper, easier to access and as effective as legal product, a view shared by patients in New Zealand [43]. In contrast, the perception that legal MC was more consistent or better in quality and safer than illicit forms was noted in American studies [51, 52], and documented in an Australian survey of MC users [8]. The message that MC is safer and more effective than recreational cannabis may be an effective public health message used to create behaviour change and move consumers from illicit to regulated MC.

### 4.4 Limitations of Study

This document analysis is limited by respondent self‐selection into the submission process. Most consumers and their carers who provided submissions were facing the consequences of serious illness, thereby restricting the generalisability of the results.

## 5 Conclusion

The present research offers a description of how health policy, the behaviours of market actors and industry characteristics intersected to create substantial barriers to access to a health technology, thereby creating multiple vulnerabilities amongst an already vulnerable population. Patients' experiences of vulnerability included having conditions untreated or treated with ineffective medicines as well as financial, legal and psychological stress. Patients responded to their vulnerability by reducing dosage and increasing the length between prescriptions, accessing community support, accessing alternative markets and increasing feelings of control through self‐education.

## Author Contributions

**Katrina Gething:** conceptualization, methodology, visualization, writing – review and editing, writing – original draft, formal analysis. **Daniel Erku:** conceptualization, supervision, writing – review and editing. **Paul Scuffham:** writing – review and editing, supervision.

## Ethics Statement

This document analysis adheres to ethical research principles by ensuring the confidentiality of all sources, avoiding unnecessary identification of individuals within the article, and only analysing publicly available materials, respecting copyright laws and interpreting findings with sensitivity to potential social and cultural contexts, while acknowledging limitations and biases inherent in the data.

## Conflicts of Interest

The authors declare no conflicts of interest.

## Supporting information

Supporting information.

## Data Availability

The data that support the findings of this study are available in Submissions ‐ Submissions received by the Committee at https://www.aph.gov.au/Parliamentary_Business/Committees/Senate/Community_Affairs/Medicinalcannabis/Submissions. These data were derived from the following resources available in the public domain: ‐ Parliament of Australia, https://www.aph.gov.au/Parliamentary_Business/Committees/Senate/Community_Affairs/Medicinalcannabis.
