# Supplementary material for: Medicinal Cannabis and Consumer Vulnerability in Australia: A Nexus of Policy and Market Factors
Source: Health Expect. 2025 Feb 10;28(1):e70176. doi: 10.1111/hex.70176 (PMC11811393; doi:10.1111/hex.70176)
Supplement: Supplementary file 1 — Supporting information. [file HEX-28-e70176-s001.docx]

**Terms of Reference**

The current barriers to patient access to medicinal cannabis in Australia, including:

(a) the appropriateness of the current regulatory regime through the Therapeutic Goods Administration (TGA) Special Access Scheme (SAS), Authorised Prescriber Scheme and clinical trials;

(b) the suitability of the Pharmaceutical Benefits Scheme for subsidising patient access to medicinal cannabis products;

(c) the interaction between state and territory authorities and the Commonwealth, including overlap and variation between state and territory schemes;

(d) Australia’s regulatory regime in comparison to international best practice models for medicinal cannabis regulation and patient access;

(e) the availability of training for doctors in the current TGA regulatory regime for prescribing medicinal cannabis to their patients;

(f) the education of doctors in the Endogenous Cannabinoid System (ECS), and the appropriateness of medicinal cannabis treatments for various indications;

(g) sources of information for doctors about uses of medicinal cannabis and how these might be improved and widened;

(h) delays in access, and the practice of product substitution, due to importation of medicinal cannabis and the shortage of Australian manufactured medicinal cannabis products;

(i) the current status of the domestic regulated medicinal cannabis industry;

(j) the impacts on the mental and physical wellbeing of those patients struggling to access medicinal cannabis through Australia’s regulatory regime;

(k) the particular barriers for those in rural and remote areas in accessing medicinal cannabis legally;

(l) the significant financial barriers to accessing medicinal cannabis treatment;

(m) the number of Australian patients continuing to rely on unregulated supply of medicinal cannabis due to access barriers and the impacts associated with that; and

(n) any related matters.

https://www.aph.gov.au/Parliamentary_Business/Committees/Senate/Community_Affairs/Medicinalcannabis/Terms_of_Reference
